# Controlled Assembly of Luminescent Lanthanide-Organic Frameworks via Post-Treatment of 3D-Printed Objects

**DOI:** 10.1007/s40820-020-00543-w

**Published:** 2020-10-31

**Authors:** Jiahui Huang, Peiyi Wu

**Affiliations:** 1 State Key Laboratory of Molecular Engineering of Polymers, Department of Macromolecular Science and Laboratory of Advanced Materials, Fudan University, Shanghai, 200433 People’s Republic of China; 2 State Key Laboratory for Modification of Chemical Fibers and Polymer Materials, College of Chemistry, Chemical Engineering and Biotechnology, Center for Advanced Low-Dimension Materials, Donghua University, Shanghai, 201620 People’s Republic of China

**Keywords:** 3D printing, Luminescent lanthanide-organic frameworks, Macroscopic assembly, In situ growth, Optical sensing

## Abstract

Complex multiscale assemblies of metal–organic frameworks are essential in the construction of large-scale optical platforms but often restricted by their bulk nature and conventional techniques. The integration of nanomaterials and 3D printing technologies allows the fabrication of multiscale functional architectures. Our study reports a unique method of controlled 3D assembly purely relying on the post-printing treatment of printed constructs. By immersing a 3D-printed patterned construct consisting of organic ligand in a solution of lanthanide ions, in situ growth of lanthanide metal–organic frameworks (LnMOFs) can rapidly occur, resulting in macroscopic assemblies and tunable fluorescence properties. This phenomenon, caused by coordination and chelation of lanthanide ions, also renders a sub-millimeter resolution and high shape fidelity. As a proof of concept, a type of 3D assembled LnMOFs-based optical sensing platform has demonstrated the feasibility in response to small molecules such as acetone. It is anticipated that the facile printing and design approach developed in this work can be applied to fabricate bespoke multiscale architectures of functional materials with controlled assembly, bringing a realistic and economic prospect.

**Electronic supplementary material:**

The online version of this article (10.1007/s40820-020-00543-w) contains supplementary material, which is available to authorized users.

## Article Highlights

- Controlled 3D assembly of luminescent lanthanide metal–organic frameworks (LnMOFs) through additive manufacturing followed by posting-printing treatment, enabling the multiscale integration in a precisely controlled and facile manner.
- 3D-printed LnMOFs objects with tunable fluorescence properties are caused by coordination and chelation of lanthanide ions, rendering a sub-millimeter resolution and high shape fidelity.
- A type of 3D assembled LnMOFs-based optical sensing platform showing response to small molecules such as acetone is presented.

**Electronic supplementary material:**

The online version of this article (10.1007/s40820-020-00543-w) contains supplementary material, which is available to authorized users.

## Graphical abstract

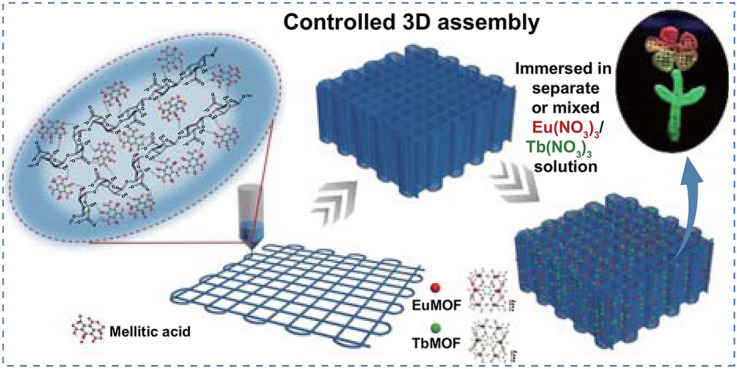

## Introduction

Metal–organic frameworks (MOFs), a versatile class of crystalline solids composed of metal clusters and organic ligands via self-assembly, have been extensively explored for their rich structural chemistry and potential applications in various areas [1–4], especially in the optical field [5–8]. Over the past years, lanthanide metal–organic frameworks (LnMOFs), as a kind of outstanding luminescent MOFs, have attracted considerable attention in optical sensing owing to their substantial Stokes shifts, sharp emission bands, high quantum yields, vivid color purity, and prolonged decay lifetime, especially those with europium (Eu) and terbium (Tb) as metal nodes [9, 10]. However, crystalline and powdery nature [11] with limited mechanical properties are not sufficient to meet the needs in the future large-scale optical applications, and traditionally manufactured devices have yet to approach the level of complexity and functional integration often exhibited in nature [9, 12–15].

To this end, the combination of nanomaterials with 3D printing technologies has achieved multiscale integration of functional nanomaterials [16–18]. Nevertheless, the granular and non-adhesive nature makes them suffer from issues like the inability of continuous flow and poor viscosity features, and unsuitable for direct use as ink to build desired macrostructures.

Recent efforts have been focused on improving printability and machinability, which is dictated by multiplexed parameters, such as the ink properties and printer hardware [19]. The most widely adopted ink formulations for 3D printing of MOFs often need additive materials, such as thermoplastic polymers [20, 21], acrylonitrile butadiene styrene [22], acrylate adhesive [23], bentonite clay [24, 25], and photopolymers [26, 27], yet requiring high processing temperature of up to 230 °C or ultraviolet curing process. Alternatively, hydrogels [28] or biomaterials [29] that are sustainable and biocompatible have been proved to hold great potential for achieving these goals. Although these strategies allow high MOF loads, the lack of controllability and flexibility with respect to loading matters is inevitable. In situ method, on the other hand, is supposed to be a promising approach for overcoming these drawbacks, especially in a precisely controlled and facile manner [30]. In most cases, a frame is used, followed by in situ step-by-step growth of MOFs on its surface [31, 32], or by post-coating a suspension of finely powdered MOFs [33]. With the combined advantages of MOFs in materials and 3D printing technique in processing, 3D-printed MOFs have been extensively exploited in water treatment [34], gas storage [22], gas adsorption [28], catalysis [23], drug delivery [29], moisture sensing [27], and batteries [35].

Nevertheless, the applications of luminescent MOFs monoliths into desirable printing patterns have only been investigated to a limited extent, and the construction of LnMOFs-based 3D optical sensing platforms with both customizable geometries and tunable fluorescence remains in the nascent stage. Due to traditional manufacture techniques such as casting [9], inkjet printing [12, 13], layer-by-layer [36], electrophoretic deposition [37], gelation [14] and others [38], luminescent MOFs are limited to 2D structures or simple 3D structures by templating, which means lack of the designability of architectures at the macroscale.

Here, we propose a strategy of controlled assembly in 3D printing based on direct ink writing (DIW), through post-treatment of the printed structures, for the construction of 3D LnMOFs-based optical platforms. We select bi-component formulations that comprised of organic ligand and commonly used alginate, providing the precursor ink with ideal and tunable rheological features through various inter/intra-molecular hydrogen bonds. Following standard printing procedures, we subject the printed constructs to immersion in individual or mixed lanthanide ions solution. Through the coordination with ligand and subsequent chelation with alginate matrix, these printed constructs are demonstrated to possess tunable emission colors with controllable in situ growth of LnMOFs and high shape fidelity. Finally, we conduct a type of optical sensing platform based on 3D LnMOFs assemblies, which is capable of detecting small molecules such as acetone. With the integrated merits of luminescent MOFs materials in optical applications and additive manufacturing technologies in processing, it not only shows the potential to open up new horizons for optical sensing but also provides a facile strategy for the versatile macroscopic assembly of functional materials with further optimizations.

## Experimental

### Materials

Europium(III) nitrate hexahydrate, terbium(III) nitrate pentahydrate, mellitic acid, and ethanol were purchased from Aladdin Chemical Co. And without further purification, acetone, methanol, isopropanol, CH_2_Cl_2_, tetrahydrofuran (THF), acetonitrile, N,N-Dimethylformamide (DMF), dimethylsulfoxide (DMSO) were purchased from Sinopharm Chemical Reagent Co. Alginic acid sodium salt whose viscosity is about 500–1000 mPa s was purchased from Adamas-beta Co.

### Preparation of the Precursor Inks

Briefly, the precursor was made up of 20 wt% alginic acid sodium salt and mellitic acid that ranges from 2 to 6 wt%. Then, the solution should be rigidly stirred for the homogenous blend at room temperature. Taking 2 wt% of mellitic acid added as an example, 1 g sodium alginate was added into 5 mL 2 wt% (0.1 g) mellitic acid aqueous solution. Then, the mixture was stirred rigidly at least 10 min to obtain the homogenous blend and named Alg-Mellitic-2, where the number 2 represents the mass ratio of mellitic acid in the precursor mixture. Similarly, Alg-Mellitic-4 and Alg-Mellitic-6 were synthesized through the same procedures except that the concentration of the mellitic aqueous solution was 4 and 6 wt%, respectively.

### 3D Printing of the Precursor Inks

The precursor was extruded and 3D-printed by a precision 3D printing system (3D Bio-Architects working station, Regenovo). The precursor scaffolds with multilayers were manufactured with an initial angle of 0° and a rotation angle of 90°. The temperature of the platform was kept at 25 °C during the printing process. According to the different viscoelastic properties of different precursor inks, we chose different 3D printing parameters. By evaluating the shear-thinning behavior (stress and viscosity as a function of different shear rates), we can first estimate the extrusion pressure range, since the suitable viscosity for 3D extrusion printing should be in the range of 0.1–50 Pa s. Then, we can choose the nozzle size, the principle of choosing a nozzle is that the ink can be smoothly squeezed through the needle. Moving speed is the last consideration. These three parameters influence each other in the printing; for example, under fixed extrusion pressure and nozzle size, fast moving speed would lead to the decrease in filament diameter. As a result, by fixing two of the parameters to adjust the other parameter, we can quickly find the optimized parameters. For Alg-Mellitic-2, the diameter of the chosen conical needle tip was 0.6 mm. With the extrusion pressure of 0.4 MPa by an air-powered dispensing system, the moving speed was 3 mm s^−1^. For Alg-Mellitic-4, the diameter of the chosen conical needle tip was also 0.6 mm. The moving speed was 4 mm^−1^ with the extrusion pressure of 0.45 MPa. For Alg-Mellitic-6, the tip diameter of 0.41 mm and an extrusion speed of 2 mm s^−1^ were chosen. The applied pressure was 0.35 MPa. When extruded, the precursor was shear-thinning and quickly recovered to high viscosity for shape retention. The 3D-printed precursor scaffolds were free-standing with high fidelity because of excellent viscoelasticity. The following preparation and characterization processes are based on the precursor ink of Alg-Mellitic-4, unless otherwise stated.

### Controlled In Situ Growth of LnMOFs with Post-printing Treatment

The 3D-printed precursor scaffolds were soaked in 20 wt% europium(III) nitrate hexahydrate ethanol/aqueous solution or 20 wt% terbium(III) nitrate pentahydrate ethanol/aqueous solution or their mixture with different volume ratios. It is worth noting that the volume ratio between ethanol and water is 3:7. The soaking time was 5 min at room temperature. Here, the amount of trivalent metal ion (Eu^3+^ or Tb^3+^) was sufficient for the in situ growth of lanthanide-organic frameworks while chelating carboxylic groups in alginic acid sodium salt for further shape retention. The finally obtained 3D LnMOFs composites were named as Alg-EuMOF-2/4/6 (or Alg-TbMOF-2/4/6), where number 2, 4, and 6 meant the weight percentage of mellitic acid in the composite matrix as mentioned above. For the filament in Fig. 4c, d, we immersed both ends in Eu^3+^ and Tb^3+^ solutions at the same time. For flower-like structure in Fig. 4e, f, we used a dropper to add different mixed metal solutions to different parts.

### Measurement of the Precursor Inks

All rheological tests of the precursor were conducted on a HAAKE MARS modular advanced rheometer with a 25-mm parallel plate at 25 °C. Dynamic frequency sweep tests were measured by an oscillation mode using a fixed oscillatory strain of 0.5%. Viscometry tests were performed with shear rates ranging from 0.01 to 100 s^−1^. Strain sweep measurements and continuous step strain measurements were performed from 0.1 to 500% and back to 0.1% strain at the frequency of 1 Hz (25 °C). Fourier transforms infrared (FTIR) spectra were characterized by Nicolet Nexus 470 spectrometer with a diamond crystal as the window material.

### Characterization for the Controlled Assembly of LnMOFs

Samples for the following measurements were in dimensions of 10 × 10 × 2 mm^3^ with the gap size of 0.9 mm and the pore wall thickness of 0.5 mm. The optical micrographs were taken by a polarized optical microscope (Leica DM2500P). The microstructures of the 3D-printed lanthanide-organic frameworks were recorded by field emission scanning electron microscopy (FESEM, Zeiss, Ultra 55). The element mapping results were observed from the energy dispersive spectrometer (EDS) equipped on a FESEM (S-4800, Hitachi, Japan). The powder X-ray diffraction (XRD) characterizations of 3D-printed LnMOFs were measured on D8 ADVANCE and DAVINCI DESIGN (Bruker) with Cu Kα radiation. Thermogravimetric analysis (TGA) was carried out on TGA PE Pyris 1 from 50 to 800 °C with a heating rate of 10 °C/min in N_2_ atmosphere. Fluorescent emission and excitation spectra were obtained with FLS (QM40) at ambient conditions. Photographs of photoluminescence were taken using a camera under excitation of 254 nm by a hand-held UV lamp.

## Results and Discussion

### Properties and Printability of the Precursor Ink

**Fig. 1:**
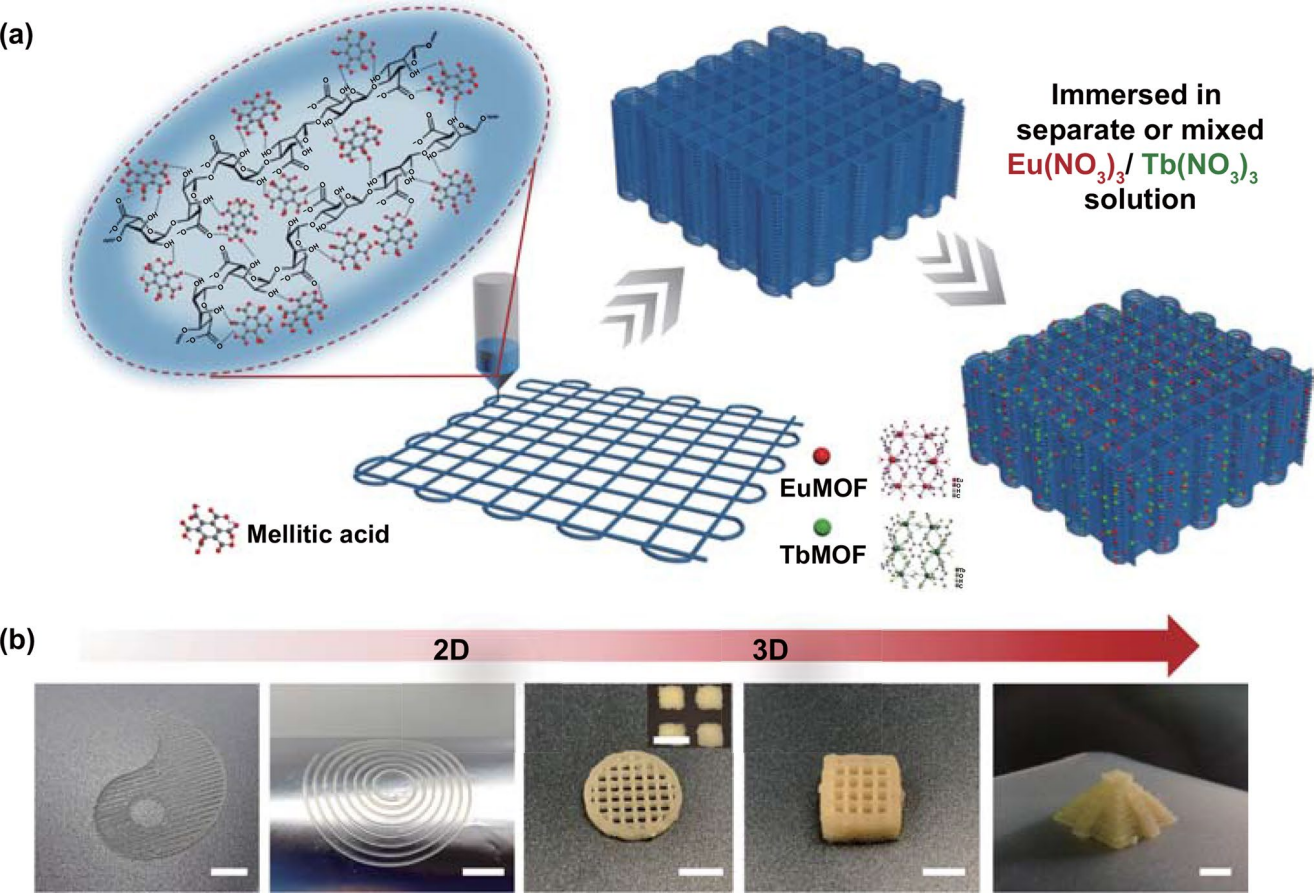
Mechanism and illustration of controlled assembly in 3D printing. **a** Schematic diagram of the 3D printing process and post-printing treatment for in situ growth of LnMOFs within the 3D-printed constructs. **b** Digital photos of the 3D-printed patterns and scaffolds (Scale bars: 5 mm). Inset is the enlarged image representing the pores of the printed structure with sub-millimeter resolution (Scale bar: 1 mm)

The process of preparing bi-component precursor inks and controllable in situ growth of LnMOFs within printed constructs is schematically shown in Fig. 1a. Firstly, mix the organic ligand mellitic acid with the sodium alginate to obtain precursor inks with favorable rheological properties. In this work, we focused on a kind of unique photoluminescent LnMOFs, Ln_2_(Mellitic)^.^6H_2_O, where Ln is Eu^3+^ or Tb^3+^, generating EuMOFs with red fluorescence or TbMOFs with green fluorescence, respectively. The ligand chosen here is mellitic acid with high connectivity, which can enhance bridges between several metal centers, take multiple coordination methods, and produce multidimensional frameworks [12]. In this regard, the rapid assembly of Ln_2_(Mellitic)·6H_2_O can take place at ambient conditions, unlike other hydrothermal or solvothermal prepared LnMOFs materials [11, 39]. Although hydrothermal or solvothermal methods can produce LnMOFs with higher purity, higher crystal structure and uniform grain size, it is not applicable to our work, since our purpose is to assemble at room temperature. Besides, the six carboxyl groups of each mellitic acid provide sufficient hydrogen bonding interactions and coordination. Sodium alginate, composed of 1,4-linked *β*-D-mannuronic acid (M-block) and *α*-L-guluronic acid (G-block), has gained popularity in 3D printing because of its appropriate rheological properties, hydrophilicity, nontoxicity, and low cost [40]. It has also been confirmed the existence of copious inter/intra-molecular hydrogen bonds in sodium alginate as well as fast ionic crosslinking capacity [41]. We consider that sodium alginate plays two important roles here. On the one hand, alginate is used as a rheological modifier due to its appropriate viscosity and shear-thinning property. When ionic crosslinks subsequently introduced, the obtained 3D-printed scaffolds could further maintain their shape and thus serve as a robust substrate for the in situ assembly of LnMOFs. On the other hand, alginate can function as binders of the ligand via molecular hydrogen bonds. Then, the ink is loaded into a matching syringe of the 3D printing work station. The bi-component combination inks could be extruded smoothly and deposited on various substrates in a layer-by-layer manner from 2D patterns to 3D scaffolds, such as Tai Ji pattern, spiral coil, ten-layer cylinder, 20-layer cube scaffold and pyramid-like structure (Fig. 1b and Movie S1). The 3D-printed objects exhibit a high sub-millimeter resolution (Table S1), maintaining the shape fidelity evaluated by the difference of the real printed construct to the related sliced CAD file (Fig. S1) [42]. Following standard printing procedures, we further subject the printed constructs to immersion in individual or mixed lanthanide ions solution for post-printing treatment. Through the coordination with ligand and subsequent chelation with alginate matrix, these printed constructs are demonstrated to possess tunable fluorescence with controllable in situ growth of LnMOFs as well as high shape fidelity.

**Fig. 2:**
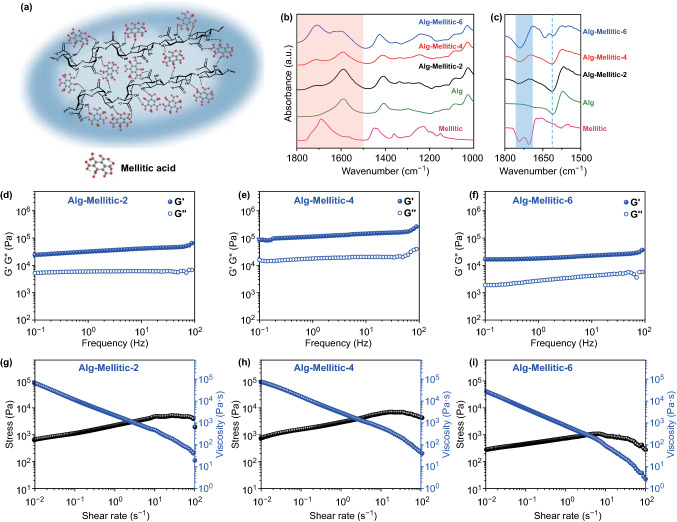
Properties and printability of the precursor inks. **a** Schematic structure of the multiple molecule interactions between ligand mellitic acid and sodium alginate. **b** FTIR spectra of the mellitic acid, sodium alginate, Alg-Mellitic-2, Alg-Mellitic-4 and Alg-Mellitic-6 in the regions of 1800–1000 cm^−1^, respectively. **c** Corresponding second derivative curves in the regions of 1800–1500 cm^−1^. **d**–**f** Storage (G′) and loss (G″) moduli of the precursor inks under different frequencies. **g**–**i** Shear-thinning behavior of the precursor inks (stress and viscosity against different shear rates)

Considering that the ligand content might affect the ink properties and ultimately affect the assembly process, we compared ink formulations and 3D LnMOFs assemblies with different ligand additions. We first investigate the rheological properties of precursor inks and surprisingly find that they can be adjusted by controlling the proportion of added ligands. The precursor ink was made up of 20 wt% sodium alginate and mellitic acid that ranges from 2 to 6 wt% by thoroughly stirring. The homogenous blend was finally obtained and named Alg-Mellitic-n, where the number n represented the mass ratio of mellitic acid in the precursor mixture. The interaction and regulation mechanism between bi-component inks have been further investigated by rheological characterization and Fourier transforms infrared (FTIR) spectroscopy. Figure 2a shows the schematic diagram of multiple hydrogen bonds between the molecules of mellitic acid and sodium alginate involving various inter/intra-molecular hydrogen bonds. The addition of different proportions of mellitic acid has great influences on the interaction of hydrogen bonds, which are correspondingly reflected in the rheological properties of the ink. According to the dynamic frequency sweep characterization (Fig. 2d–f), the storage modulus (G′) is always higher than the loss modulus (G″) in all precursor inks, indicating their highly viscoelastic state. The increase in mellitic acid percentage leads to an upward and then downward trend in both G′ and G″, since the rheological response is intimately coupled with the material structure. This effect is confirmed by a series of FTIR spectra (Figs. 2b, c and S2). The COO^−^ stretching band of sodium alginate is located at 1610 cm^−1^, which also appears in all inks. As for mellitic acid, 1706 and 1745 cm^−1^ of *v*(COOH) correspond to C=O stretching modes of cyclic hydrogen-bonded carboxylic acid in dimeric form and free ester carbonyl, respectively [43]. The C=O stretching band of inks with different formulations is located at the medium frequency, indicating the disruption of strong hydrogen bonds in mellitic acid and the formation of dynamic hydrogen bonds. Specifically, the C=O absorption peak of Alg-Mellitic-2, Alg-Mellitic-4 and Alg-Mellitic-6, after shifting to lower wavenumber, is located at 1743, 1737, and 1742 cm^−1^, respectively. The shift of wavenumber from 1743 to 1737 cm^−1^ indicates the enhancement of hydrogen bond interaction in Alg-Mellitic-4, which corresponds to the improvement of modulus (G′ and G″). On the contrary, Alg-Mellitic-6 shows higher intensity at the C=O stretching band, mainly due to the conversion of sodium alginate to alginic acid in acid medium. There also appears an extra band at 1641 cm^−1^ of Alg-Mellitic-6, which is presumably attributed to the partial conversion of COOH on mellitic acid to COO^−^, that is, ionized. The ionization of mellitic acid can promote coordination with lanthanide ions. Compared with the first two, the low modulus, as well as the reduced viscosity of Alg-Mellitic-6, is reflected in the rheological measurements. Overall, tunable printability of ink formulations is attributed to molecular interactions.

Moreover, all the precursor inks exhibit a pronounced shear-thinning behavior (Fig. 2g–i) and are promising candidates for 3D printing. For the extrusion-based direct ink writing (DIW) technique, the shear-thinning behavior under extrusion and the ability to keep good shape after extrusion of inks are prerequisites that improve printability and fidelity [44]. Here, we take Alg-Mellitic-4 for example, and the analysis details of the remaining two recipes are in the supplementary information. As shown in Fig. 2h, at a low shear rate (about 0.1 s^−1^), the viscosity is about 16,920 Pa s and the system exhibits solid-like properties, whereas at a high shear rate (about 100 s^−1^), the viscosity decreases to around 40 Pa s showing liquid-like properties, which is suitable for direct ink writing. In the high-strain rate region of 1 to 100 s^−1^, we could quantitatively evaluate the shear-thinning behavior of Alg-Mellitic-4 via the power-law fluid model, τ = *K* $\dot{\gamma }$^*n*^, where *K* and *n* refer to consistency index and exponent, respectively. By fitting, the exponent *n* is calculated to be 0.24. Then, when the power-law fluid flows through a cylindrical tube of radius *r*, the shear rate $\dot{\gamma }$ is predictable via the Rabinowitsch–Mooney equation. Thus the corresponding viscosity is calculated to be about 46 Pa s (Fig. 2h), which is in the suitable range for direct ink writing [45, 46]. Once squeezed, it can recover the solid-like behavior for shape retention and high fidelity. The recovery behavior can also be evaluated by strain sweep measurements of the precursor ink from 0.1% to 500% and back to 0.1% strain, displaying the storage modulus (G′) and loss modulus (G″) as a function of shear strain and good recoverability under high shear strain (Fig. S3a). Continuous step-strain measurements at a high-amplitude oscillatory (*γ* = 0.1%) and a low-amplitude oscillatory (*γ* = 100%) can further demonstrate the fast reconfiguration and the thixotropic property of inks (Fig. S3b). Once the inks are extruded, the applied shear strains (or stresses) disappear, and the ink can return to the original solid-like state immediately. Consequently, it is possible to successfully fabricate 3D architecture by the continuous extrusion of bi-component inks layer-by-layer following different structural designs.

### In Situ Growth of 3D-Printed LnMOFs with Tunable Fluorescence

**Fig. 3:**
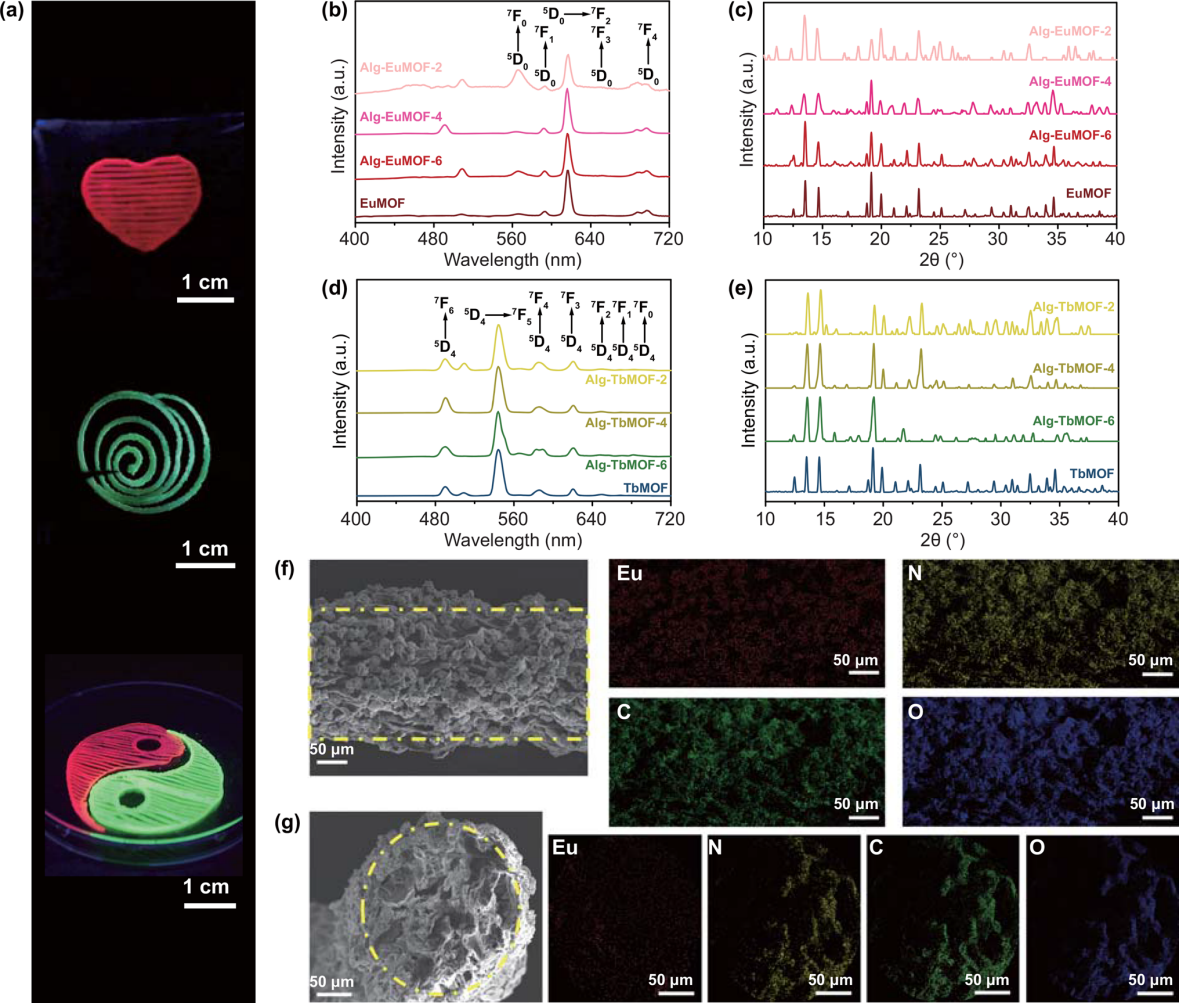
Post-printing treatment of 3D-printed constructs in the individual lanthanide ion solution. **a** Photographs of several 3D patterns under UV light irradiation (*λ*_exc_ = 254 nm). **b, c** Fluorescence emission spectra and XRD patterns of Alg-EuMOF with different mellitic acid additions. **d**, **e** Fluorescence emission spectra and XRD patterns of Alg-TbMOF with different mellitic acid additions. **f** Side SEM image and **g** section SEM image of Alg-EuMOF-4 and the corresponding energy dispersive spectrometer (EDS) mapping of Eu, N, C, and O elements

Following the extrusion printing process with the above-mentioned inks, we subject the printed constructs to immersion in either individual or mixed lanthanide ions solution, both of which have been demonstrated the controlled assembly of LnMOFs. We first investigate post-printing treatment of 3D-printed constructs in individual lanthanide ions solution, and meanwhile the effect of different ligand content. The obtained 3D LnMOFs composites were named as Alg-EuMOF-2/4/6 or Alg-TbMOF-2/4/6, depending on the immersion of lanthanide ions solution (Eu^3+^ or Tb^3+^). Notably, among the mixed solvent, the optimized volume ratio of water and ethanol is 7:3 [12]. The appropriate amount of ethanol molecules plays a key role in the formation of LnMOFs crystals, which is also widely adopted in other nucleation and assembly systems [47, 48]. In our work, the ethanol molecules could completely or partially replace the solvent precursor around the building blocks and promote the rapid nucleation of crystals. Simultaneously, the addition of ethanol also has a positive impact on the homogeneous crosslinking of alginate in the lanthanide ions solution [49]. In Fig. 3a, various 3D patterns with vivid emission colors after immersed in the corresponding metal solutions are presented under an ultraviolet lamp (*λ*_exc_ = 254 nm), such as red heart, green spiral coil and red and green Taiji pattern, which bear out the in situ growth of LnMOFs within sodium alginate filaments (Fig. S4). Because sodium alginate has almost no fluorescence at the excitation wavelength of 254 nm, which does not overlap with the emission wavelength of EuMOFs or TbMOFs [50]. Therefore, fluorescent spectra and XRD were immediately performed to confirm the assembly of LnMOFs crystals and their emission properties. The pristine EuMOFs and TbMOFs crystals with ethanol as precipitant agents are about 20 μm in size, as shown in SEM images (Fig. S5). Compared with pristine EuMOFs and TbMOFs, fluorescent emission spectra and XRD show the successful synthesis of EuMOFs and TbMOFs in all cases. Figure 3b, d shows the typical narrow characteristic emissions of EuMOFs at 615 nm and TbMOFs at 545 nm corresponding to the Eu^3+ 5^D_0_ → ^7^F_2_ and Tb^3+ 5^D_4_ → ^7^F_5_ transitions, respectively. It turned out that the ligand could role as the sensitizers for the lanthanide ions based on the antenna effect, that is, the coordinated ligands can absorb the incident radiation and the energy transfers from the ligand to the Eu^3+^ and Tb^3+^ center effectively [6]. This result is further confirmed by XRD analyses (Fig. 3c, e), in which the 3D LnMOFs scaffolds are indeed well-formed and iso-structural to those previously reported [51]. In the immersion process, trivalent ions also interact with three carboxylic groups of different alginate chains at the same time, forming a three-dimensional valent structure and a compact network [52, 53], as shown in Fig. S6. Compared with divalent cations, the binding extent of trivalent ions with alginate is enhanced. Considering this aspect, the solutions we used is high concentration and excessive for immersion, which ensures either the formation of LnMOFs or the crosslinking of sodium alginate. In addition, The volume shrinkage of the prints is estimated at 37.0% (Fig. S7), mainly caused by the crosslinking of lanthanide ions with alginate and solvent exchange with ethanol. Therefore, fluorescent emission spectra and XRD reveal the excellent fluorescence performance of 3D LnMOFs structures after the post-treatment with individual lanthanide ions.

Furthermore, the micromorphology of in situ assembled LnMOFs within the sodium alginate scaffold was characterized by SEM. SEM images confirm the controlled assembly of LnMOFs among sodium alginate filaments, and corresponding element mapping results show that the europium ions (europium element) and EuMOFs (nitrogen element) are uniformly decorated on the surface (Fig. 3f) and distributed inside of the sodium alginate (Fig. 3g), showing a MOF-rich area in its external part. As the printed objects immersed in Eu^3+^ or Tb^3+^ solutions, traction forces exerted by Eu^3+^ or Tb^3+^ ions drive the alginate motion outward and then form chelation with these ions [54], due to the various inter/intra-molecular hydrogen bonds between ligand and alginate, during which more ligands are exposed at the periphery. This ensures that the functional LnMOFs growing in situ tend to be anchored in the outer layer and not be encapsulated by the polymer matrix, which also explains the excellent fluorescence performance of 3D LnMOFs structures after the post-treatment. When the addition of ligand increases, the assembly of EuMOFs or TbMOFs becomes denser (Figs. S8 and S9). Meanwhile, rougher structures of Alg-EuMOF-6 and Alg-TbMOF-6 are caused by the conversion of sodium alginate to alginic acid, and the reduced crosslinks between sodium alginate and lanthanide ions. This phenomenon is also consistent with the weight loss of TGA analysis (Fig. S10). Before 200 °C, it was mainly attributed to the loss of combined water and free water. The EuMOFs remains stable during the heating process and does not thermally decompose until 550 °C, at which sodium alginate is almost completely decomposed. As such, we roughly estimate the load of EuMOFs with the weight loss after 550 °C, which shows a significant increase from 5.76% to 15.66% with the increase in ligand from 2 to 6 wt%. This is probably because the higher concentration of the ligand increases the site of binding to the metal ion, thereby leading to denser and larger crystal assembly. Notably, gradually growing the LnMOFs into the printed architecture has enhanced the mechanical properties of the hybrids instead of making the object brittle and not self-supportive (Fig. S11). This is because by soaking the lanthanide metal ions, the printed alginate structure is also crosslinked in the process of in situ growing LnMOFs, thereby contributing to high fidelity.

**Fig. 4:**
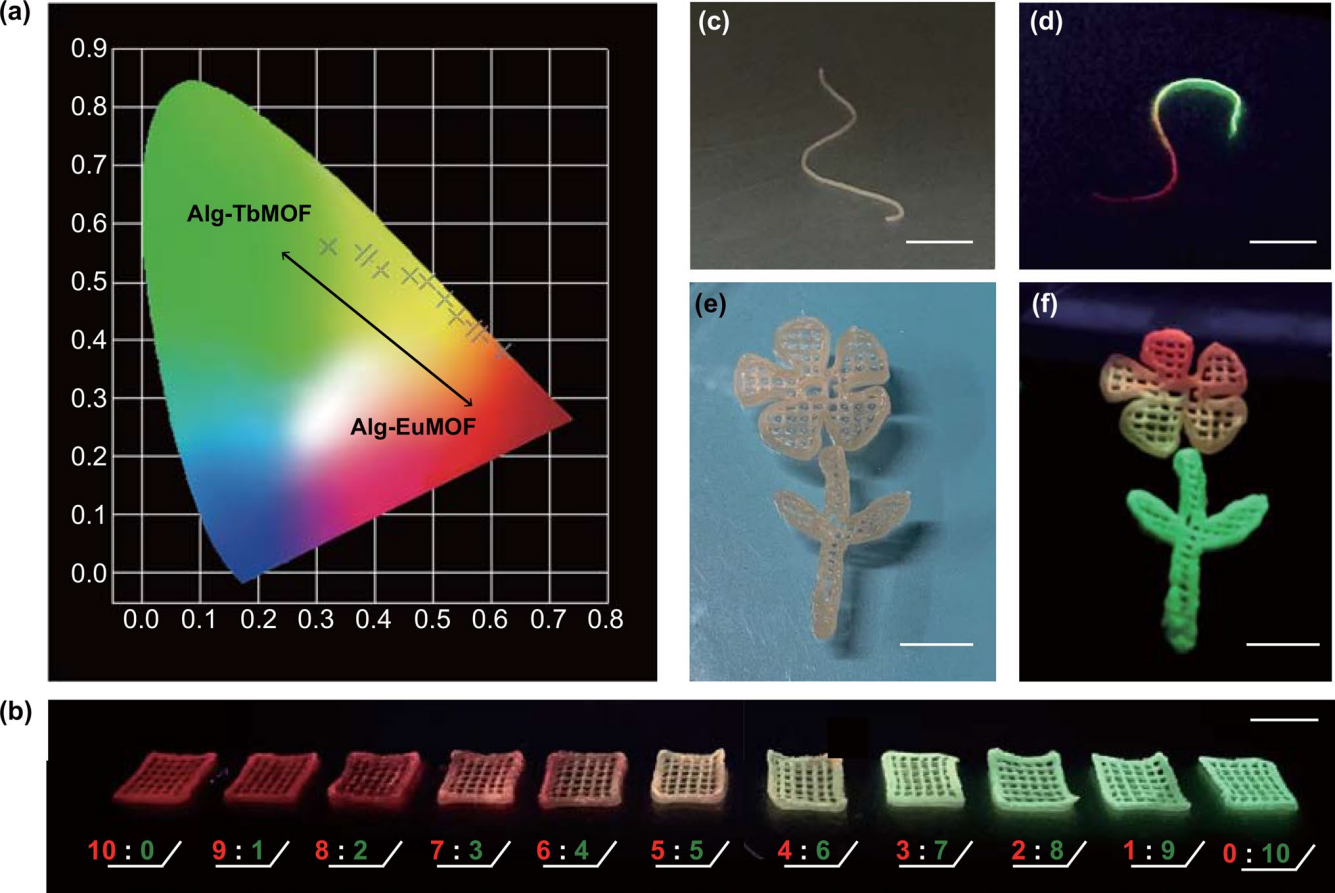
Post-printing treatment of 3D-printed constructs in the mixed lanthanide ions solution. **a** CIE 1931 chromaticity diagram for Alg-TbMOF and Alg-EuMOF and mixed metal Alg-Eu/TbMOF (from left to right: Eu^3+^/Tb^3+^ = 10:0, 9:1, 8:2, 7:3, 6:4, 5:5, 4:6, 3:7, 2:8, 1:9, 0:10) and **b** the corresponding luminescent images of 3D-printed constructs under UV irradiation (*λ*_exc_ = 254 nm). **c**–**f** Photographs of filament and 3D-printed flower-like structure that can be post-designed with tunable fluorescence in different positions originating from mixed lanthanide ions. Scale bars are 1 cm

In addition, benefitting from the adjustable ratio of Eu^3+^ and Tb^3+^ ions in the metal solution, we have successfully prepared 3D assemblies with mixed-metal LnMOFs, whose photoluminescence properties can be fine-tuned. We also proved their excellent and tunable emission performance through fluorescent emission spectra. When varying the ratio of Eu^3+^ and Tb^3+^ ions from 10:0 to 0:10, the intensity of the characteristic emissions of EuMOFs gradually decreased and that of TbMOFs gradually enhanced (Fig. S12). Correspondingly, the Commission Internationale de L’Eclairage 1931 (CIE) chromaticity diagram coordinates also transform from the red zone through the yellow zone and finally to the green zone (Fig. 4a). The relevant optical images are given in Fig. 4b, 3D constructs here were in dimensions of 10 × 10 × 2 mm^3^ with the gap size of 0.9 mm and the wall thickness of 0.5 mm. Therefore, taking advantage of this post-printing treatment method, we can synthesize specific LnMOFs within customizable 3D architectures on-demand to acquire the desired macroscopic assemblies and fluorescent properties, which shows the potential application in anti-counterfeiting marks or invisible security labeling, utilizing adjustable emission colors as information storage unit (Fig. S13).

More interestingly, this strategy offers more flexibility and diversity in the choice of assembled materials. To be specific, selectively immersed in different lanthanide ions to generate different mixed-metal LnMOFs among different positions of 3D-printed architectures, leading to multiple fluorescent colors on a single assembly. For example, the two ends of the extruded fiber (Fig. 4c, d) can show a gradual change from green to red fluorescent color under an ultraviolet lamp (*λ*_exc_ = 254 nm). Besides, the complex 3D flower-like structure can also be assigned multiple fluorescence colors in different parts of petals and leaves (Fig. 4e, f). The entire assembly processes took place at room temperature without cumbersome processing steps. To justify the successful assembly of LnMOFs onto the matrix, we took polarized optical micrographs (POM) under crossed polarizers. In Fig. S14, the areas where LnMOFs were generated in mass production exhibited distinct light, confirming the uniform growth of LnMOFs crystals among the 3D scaffolds. However, inevitably, the accuracy of spatial programming in assigning multiple colors to a 3D object has a relatively large dependence on the printing structure (e.g., different parts are preferably partly continuous or separated), otherwise it would appear a smooth transition between the two properties. This limitation could be improved through the design of the print structure to improve the controllability and accuracy of fluorescence color.

In summary, all these results indicate the flexibility and controllability of this printing strategy, and meanwhile the 3D assemblies retain the superior luminescent performance of LnMOFs. These merits may promise more potential in future optical devices.

### Potential Optical Applications

**Fig. 5:**
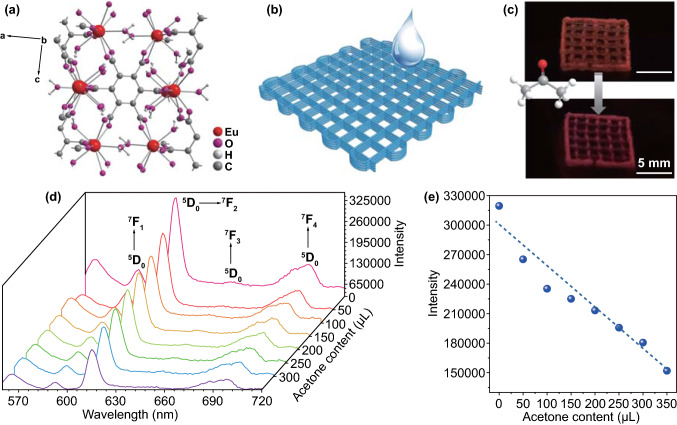
Proof-of-concept demonstration of optical sensing platform. **a** Molecular structure of EuMOF obtained from single-crystal crystallography. **b** 3D-printed architecture of the optical sensing system. **c** Photographs of fluorescence quenching can be seen by the naked eye in the case of colorimetric response. **d** Photoluminescence spectra of Alg-EuMOF-4 and **e** the corresponding ^5^D_0_ → ^7^F_2_ transition intensity changes in response to various content acetone (*λ*_exc_ = 254 nm)

Finally, to expand its conceptual feasibility, we conduct a type of optical sensing platform based on 3D LnMOFs assemblies. By making use of the luminescent open metal site, these fluorescent scaffolds with different emissions can be exploited as a detector of small molecules such as acetone [55]. Figure 5a depicts a unit cell of the EuMOFs crystal that has been reported in detail before [51]. The resulting framework exists a 2D net constituted with the aromatic rings and the Ln^3+^ ions stacked on the *bc* plane, and also 1D channels along the *c*-axis. These channels are capped by free water molecules and terminals [51, 55]. We found that the addition of acetone to the abovementioned scaffold printed in dimensions of 10 × 10 × 2 mm^3^ caused an evident decline of its fluorescence intensity. When the content of acetone added was only 50 μL, the fluorescent color of the scaffold under ultraviolet light was visually reduced by the naked eye in the case of colorimetric response (Fig. 5b, c). As shown in the fluorescence spectra of Fig. 5d, it is remarkable that the luminescence intensity of Eu^3+ 5^D_0_ → ^7^F_2_ transition at 615 nm decreases versus the volume content, which could be well-fitted with a linear decay, giving an estimated detection limit of 3.83 μL (Figs. 5e and S15). These results are also in line with previous studies and could be explained by luminescent open Eu^3+^ sites [55]. It is expected that the binding interaction of open sites of luminescent metals with guest solvent molecules makes an important impact [55]. As the acetone content increases, the weakly coordinated ethanol and free water molecules on the Eu^3+^ sites are gradually replaced by acetone molecules, thus leading to fluorescence attenuation. The response speed is fast, as shown in Fig. S16. Besides, it exhibits selectivity on acetone quenching and the other small amounts of solvents have no obvious effect on the luminescence quenching properties of acetone (Fig. S17). The quenching performance was regained after removing acetone by rinsing with mixed solvent (the volume ratio of ethanol and water is 3:7) three times, showing the reversibility for several cycles (Fig. S18). The selectivity, reversibility and response rate of our 3D scaffold are comparable to those reported sensors for acetone (Table S2), while the sensitivity can be improved. This may ascribe to the pores in the sodium alginate scaffold that would also absorb part of the solvent molecules and those EuMOFs growing inside alginate, resulting in a reduced degree of fluorescence quenching. Overall, this visual and designable sensory system will certainly play a pivotal part in prospective optical fields with the combined merits of luminescent MOFs materials in optical applications and 3D printing technique in processing.

## Conclusions

In summary, to address the limitations of bulk luminescent MOFs in future large-scale processing and application in optical fields, we develop a unique method of controlled assembly purely relying on the post-printing treatment of printed constructs. By immersing a 3D-printed patterned construct consisting of the organic ligand in the solution of lanthanide ions, in situ growth of LnMOFs can rapidly occur, resulting in macroscopic assemblies and tunable fluorescence properties. We also endeavor on a proof-of-concept in optical sensing and prove its performance as devices. We therefore anticipate the widespread adoption of our unique technology in the future large-scale controlled assembly of bulk functional materials for various application areas with further optimizations.

## Electronic supplementary material

Below is the link to the electronic supplementary material.

Supplementary file 1 (PDF 1580 kb)

Supplementary file 2 (MP4 2659 kb)

## References

[1] Liu G, Chernikova V, Liu Y, Zhang K, Belmabkhout Y (2018). Mixed matrix formulations with mof molecular sieving for key energy-intensive separations. Nat. Mater..

[2] Yuan S, Feng L, Wang K, Pang J, Bosch M (2018). Stable metal–organic frameworks: design, synthesis, and applications. Adv. Mater..

[3] Hong X-J, Song C-L, Yang Y, Tan H-C, Li G-H, Cai Y-P, Wang H (2019). Cerium based metal–organic frameworks as an efficient separator coating catalyzing the conversion of polysulfides for high performance lithium–sulfur batteries. ACS Nano.

[4] Sun Y, Zheng L, Yang Y, Qian X, Fu T (2020). Metal–organic framework nanocarriers for drug delivery in biomedical applications. Nano-Micro Lett..

[5] Schoedel A, Li M, Li D, O’Keeffe M, Yaghi OM (2016). Structures of metal–organic frameworks with rod secondary building units. Chem. Rev..

[6] Hu Z, Deibert BJ, Li J (2014). Luminescent metal–organic frameworks for chemical sensing and explosive detection. Chem. Soc. Rev..

[7] Cui Y, Zhang J, He H, Qian G (2018). Photonic functional metal–organic frameworks. Chem. Soc. Rev..

[8] Dong J, Zhao D, Lu Y, Sun W-Y (2019). Photoluminescent metal–organic frameworks and their application for sensing biomolecules. J. Mater. Chem. A.

[9] Su Y, Yu J, Li Y, Phua SFZ, Liu G (2018). Versatile bimetallic lanthanide metal–organic frameworks for tunable emission and efficient fluorescence sensing. Commun. Chem..

[10] Boroujerdi R, Abdelkader A, Paul R (2020). State of the art in alcohol sensing with 2d materials. Nano-Micro Lett..

[11] Yang X, Lin X, Zhao Y, Zhao YS, Yan D (2017). Lanthanide metal–organic framework microrods: colored optical waveguides and chiral polarized emission. Angew. Chem. Int. Ed..

[12] da Luz LL, Milani R, Felix JF, Ribeiro IRB, Talhavini M (2015). Inkjet printing of lanthanide–organic frameworks for anti-counterfeiting applications. ACS Appl. Mater. Interfaces.

[13] Wang Y-M, Tian X-T, Zhang H, Yang Z-R, Yin X-B (2018). Anticounterfeiting quick response code with emission color of invisible metal–organic frameworks as encoding information. ACS Appl. Mater. Interfaces.

[14] Chen F, Wang Y-M, Guo W, Yin X-B (2019). Color-tunable lanthanide metal–organic framework gels. Chem. Sci..

[15] Chen M, Hu X, Li K, Sun J, Liu Z (2020). Self-assembly of dendritic-lamellar mxene/carbon nanotube conductive films for wearable tactile sensors and artificial skin. Carbon.

[16] Elder B, Neupane R, Tokita E, Ghosh U, Hales S, Kong YL (2020). Nanomaterial patterning in 3d printing. Adv. Mater..

[17] Deng S, Wu J, Dickey MD, Zhao Q, Xie T (2019). Rapid open-air digital light 3d printing of thermoplastic polymer. Adv. Mater..

[18] Doan HV, Amer Hamzah H, Karikkethu Prabhakaran P, Petrillo C, Ting VP (2019). Hierarchical metal–organic frameworks with macroporosity: Synthesis, achievements, and challenges. Nano-Micro Lett..

[19] Gong J, Schuurmans CCL, van Genderen AM, Cao X, Li W (2020). Complexation-induced resolution enhancement of 3d-printed hydrogel constructs. Nat. Commun..

[20] Evans KA, Kennedy ZC, Arey BW, Christ JF, Schaef HT, Nune SK, Erikson RL (2018). Chemically active, porous 3d-printed thermoplastic composites. ACS Appl. Mater. Interfaces.

[21] Pei P, Tian Z, Zhu Y (2018). 3d printed mesoporous bioactive glass/metal–organic framework scaffolds with antitubercular drug delivery. Microporous Mesoporous Mater..

[22] Bible M, Sefa M, Fedchak JA, Scherschligt J, Natarajan B, Ahmed Z, Hartings MR (2018). 3d-printed acrylonitrile butadiene styrene-metal organic framework composite materials and their gas storage properties. 3D Print. Addit. Manuf..

[23] Young AJ, Guillet-Nicolas R, Marshall ES, Kleitz F, Goodhand AJ (2019). Direct ink writing of catalytically active UIO-66 polymer composites. Chem. Commun..

[24] Thakkar H, Eastman S, Al-Naddaf Q, Rownaghi AA, Rezaei F (2017). 3d-printed metal–organic framework monoliths for gas adsorption processes. ACS Appl. Mater. Interfaces.

[25] Lefevere J, Claessens B, Mullens S, Baron G, Cousin-Saint-Remi J, Denayer JFM (2019). 3d-printed zeolitic imidazolate framework structures for adsorptive separations. ACS Appl. Nano Mater..

[26] Halevi O, Tan JMR, Lee PS, Magdassi S (2018). Hydrolytically stable mof in 3d-printed structures. Adv. Sustain. Syst..

[27] Maldonado N, Vegas VG, Halevi O, Martínez JI, Lee PS (2019). 3d printing of a thermo- and solvatochromic composite material based on a Cu(ii)–thymine coordination polymer with moisture sensing capabilities. Adv. Funct. Mater..

[28] Thakkar H, Al-Naddaf Q, Legion N, Hovis M, Krishnamurthy A, Rownaghi AA, Rezaei F (2018). Adsorption of ethane and ethylene over 3d-printed ethane-selective monoliths. ACS Sustain. Chem. Eng..

[29] Sultan S, Abdelhamid HN, Zou X, Mathew AP (2019). Cellomof: Nanocellulose enabled 3d printing of metal–organic frameworks. Adv. Funct. Mater..

[30] Zhu H, Zhang Q, Zhu S (2016). Alginate hydrogel: a shapeable and versatile platform for in situ preparation of metal–organic framework–polymer composites. ACS Appl. Mater. Interfaces.

[31] Shi Z, Xu C, Chen F, Wang Y, Li L, Meng Q, Zhang R (2017). Renewable metal–organic-frameworks-coated 3d printing film for removal of malachite green. RSC Adv..

[32] Wang Z, Wang J, Li M, Sun K, Liu C-J (2015). Three-dimensional printed acrylonitrile butadiene styrene framework coated with cu-btc metal–organic frameworks for the removal of methylene blue. Sci. Rep..

[33] Figuerola A, Medina DAV, Santos-Neto AJ, Cabello CP, Cerdà V, Palomino GT, Maya F (2019). Metal–organic framework mixed-matrix coatings on 3d printed devices. Appl. Mater. Today.

[34] Pei R, Fan L, Zhao F, Xiao J, Yang Y (2019). 3d-printed metal–organic frameworks within biocompatible polymers as excellent adsorbents for organic dyes removal. J. Hazard Mater..

[35] Lyu Z, Lim GJH, Guo R, Kou Z, Wang T (2019). 3d-printed mof-derived hierarchically porous frameworks for practical high-energy density li–o2 batteries. Adv. Funct. Mater..

[36] Marets N, Kanno S, Ogata S, Ishii A, Kawaguchi S, Hasegawa M (2019). Lanthanide-oligomeric brush films: from luminescence properties to structure resolution. ACS Omega.

[37] Feng J-F, Gao S-Y, Liu T-F, Shi J, Cao R (2018). Preparation of dual-emitting ln@uio-66-hybrid films via electrophoretic deposition for ratiometric temperature sensing. ACS Appl. Mater. Interfaces.

[38] Wang Z, Ananias D, Carné-Sánchez A, Brites CDS, Imaz I (2015). Lanthanide–organic framework nanothermometers prepared by spray-drying. Adv. Funct. Mater..

[39] Cui Y, Yue Y, Qian G, Chen B (2012). Luminescent functional metal–organic frameworks. Chem. Rev..

[40] Lin Z, Wu M, He H, Liang Q, Hu C (2019). 3d printing of mechanically stable calcium-free alginate-based scaffolds with tunable surface charge to enable cell adhesion and facile biofunctionalization. Adv. Funct. Mater..

[41] Hou L, Wu P (2019). Exploring the hydrogen-bond structures in sodium alginate through two-dimensional correlation infrared spectroscopy. Carbohydr. Polym..

[42] Ahlfeld T, Guduric V, Duin S, Akkineni AR, Schütz K (2020). Methylcellulose—a versatile printing material that enables biofabrication of tissue equivalents with high shape fidelity. Biomater. Sci..

[43] Dong J, Ozaki Y, Nakashima K (1997). Infrared, raman, and near-infrared spectroscopic evidence for the coexistence of various hydrogen-bond forms in poly(acrylic acid). Macromolecules.

[44] Rafiee M, Farahani RD, Therriault D (2020). Multi-material 3d and 4d printing: a survey. Adv. Sci..

[45] Hong S, Sycks D, Chan HF, Lin S, Lopez GP (2015). 3d printing of highly stretchable and tough hydrogels into complex, cellularized structures. Adv. Mater..

[46] Tian K, Bae J, Bakarich SE, Yang C, Gately RD (2017). 3d printing of transparent and conductive heterogeneous hydrogel–elastomer systems. Adv. Mater..

[47] Vega-Poot AG, Rodríguez-Gattorno G, Soberanis-Domínguez OE, Patiño-Díaz RT, Espinosa-Pesqueira M, Oskam G (2010). The nucleation kinetics of ZnO nanoparticles from ZnCl_2_ in ethanol solutions. Nanoscale.

[48] Zhuang J-L, Ceglarek D, Pethuraj S, Terfort A (2011). Rapid room-temperature synthesis of metal–organic framework hkust-1 crystals in bulk and as oriented and patterned thin films. Adv. Funct. Mater..

[49] Li J, He J, Huang Y, Li D, Chen X (2015). Improving surface and mechanical properties of alginate films by using ethanol as a co-solvent during external gelation. Carbohydr. Polym..

[50] Fertah M, Belfkira A, Dahmane EM, Taourirte M, Brouillette F (2017). Extraction and characterization of sodium alginate from moroccan laminaria digitata brown seaweed. Arab. J. Chem..

[51] Rodrigues MO, Paz FAA, Freire RO, de Sá GF, Galembeck A (2009). Modeling, structural, and spectroscopic studies of lanthanide-organic frameworks. J. Phys. Chem. B.

[52] Yang CH, Wang MX, Haider H, Yang JH, Sun J-Y (2013). Strengthening alginate/polyacrylamide hydrogels using various multivalent cations. ACS Appl. Mater. Interfaces.

[53] Wang MX, Yang CH, Liu ZQ, Zhou J, Xu F (2015). Tough photoluminescent hydrogels doped with lanthanide. Macromol. Rapid Commun..

[54] He C, Ye T, Teng W, Fang Z, Ruan W-S (2019). Bioinspired shear-flow-driven layer-by-layer in situ self-assembly. ACS Nano.

[55] Chen B, Yang Y, Zapata F, Lin G, Qian G, Lobkovsky EB (2007). Luminescent open metal sites within a metal–organic framework for sensing small molecules. Adv. Mater..

