# Supplementary material for: Controlled Assembly of Luminescent Lanthanide-Organic Frameworks via Post-Treatment of 3D-Printed Objects
Source: Nanomicro Lett. 2020 Oct 31;13:15. doi: 10.1007/s40820-020-00543-w (PMC8187549; doi:10.1007/s40820-020-00543-w)
Supplement: Supplementary file 1 — (PDF 1580 kb) [file 40820_2020_543_MOESM1_ESM.pdf]

Supporting Information for

Controlled Assembly of Luminescent Lanthanide-Organic Frameworks *via* Post-Treatment of 3D Printed Objects

Jiahui Huang[1], Peiyi Wu[1, 2, *]

[1]State Key Laboratory of Molecular Engineering of Polymers, Department of Macromolecular Science and Laboratory of Advanced Materials, Fudan University, Shanghai 200433, People's Republic of China

[2]State Key Laboratory for Modification of Chemical Fibers and Polymer Materials, College of Chemistry, Chemical Engineering and Biotechnology, Center for Advanced Low-Dimension Materials, Donghua University, Shanghai 201620, People's Republic of China

*Corresponding author. E-mail: peiyiwu@fudan.edu.cn (Peiyi Wu)

S1 Rheological Analysis of the 3D Printing Process

To analyse the rheological behaviors of different precursor formulas during the process of 3D printing, we performed the measurements about stress and viscosity with respect to shear rates. All the precursor inks exhibit a pronounced shear-thinning behavior (Fig. 2g-i) and are promising candidates for 3D printing. We applied different parameters for 3D printing on precursor inks with different rheological properties, and all of them can be analyzed by power-law fluid model. In the region of 1-100 s^{-1}, we applied a power-law fluid model to describe the shear stress as a function of shear rates, $\tau = K \cdot \dot{\gamma}^n$, where K and n refer to consistency index and exponent, respectively. Take Alg-Mellitic-4 as example (Fig. 2h), the exponent n of Alg-Mellitic-4 is calculated to be 0.24 by using a power-law fit. Then, when the power-law fluid flowing through a cylindrical tube of radius r we could perform the Rabinowitsch-Mooney equation to predict the shear rate $\dot{\gamma}$, $\dot{\gamma} = (\frac{3n+1}{4n})\frac{4Q}{\pi r^3}$, where Q refers to the volumetric flow rate. The tip diameter of 0.6 mm and an extrusion speed of 4 mm s^{-1} were chosen in this system. Calculation by the above equation, $\dot{\gamma}$ is about 95.6 s^{-1}. Thus the corresponding viscosity from Fig. 2h is about 46 Pa s. Immediately after extrusion and assuming a shear rate of far below 1 s^{-1} , the viscosity increases to > 16,900 Pa s, and hence allows for shape fidelity and post-printing stability.

Likewise, the rheological behaviours of Alg-Mellitic-2 and Alg-Mellitic-6 during the 3D printing process could also be explained. For Alg-Mellitic-2, the shear-thinning exponent n is calculated to be 0.2 and then by performing the Rabinowitsch-Mooney equation, the shear rate $\dot{\gamma}$ is predicted to be 80 s^{-1}. Finally, the corresponding viscosity of the precursor ink from Fig. 2g is about 65 Pa s, showing liquid-like

properties that can be extruded easily. The viscosity could recover to > 4500 Pa s for the shape retention upon exiting. For Alg-Mellitic-6, the shear-thinning exponent n is calculated to be 0.15, and then by performing the Rabinowitsch-Mooney equation, the shear rate γ is predicted to be 93.65 s^{-1}. Thus the relevant viscosity from Fig. 2i is about 3 Pa s that can be extruded from the needle readily. After extrusion, the viscosity is able to recover to 338 Pa s.

S2 Supplementary Figures and Tables

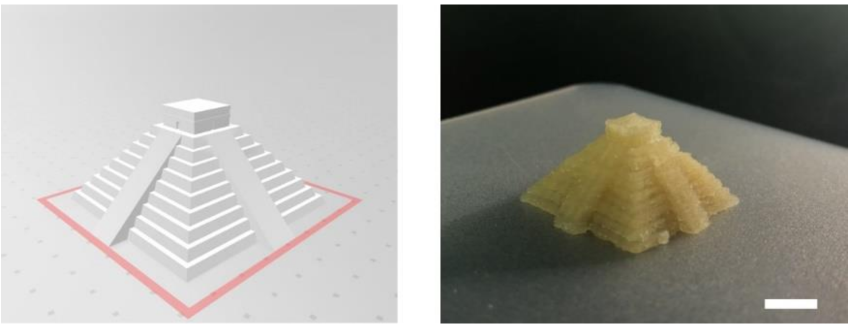

Fig. S1 Evaluation of shape fidelity by comparing the model files and our printed constructs (Scale bars: 5 cm)

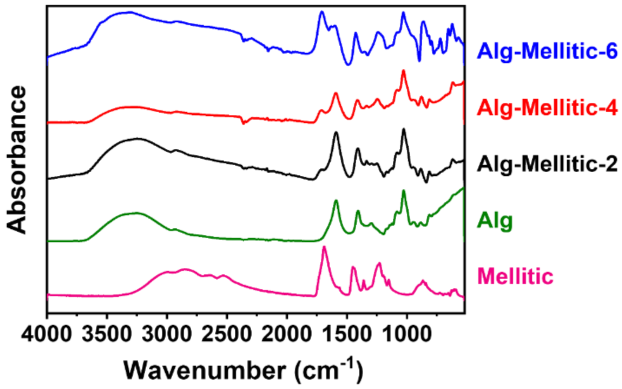

Fig. S2 FTIR spectra of the mellitic, sodium alginate, Alg-Mellitic-2, Alg-Mellitic-4, and Alg-Mellitic-6 in the regions of 4000-500 cm^{-1}

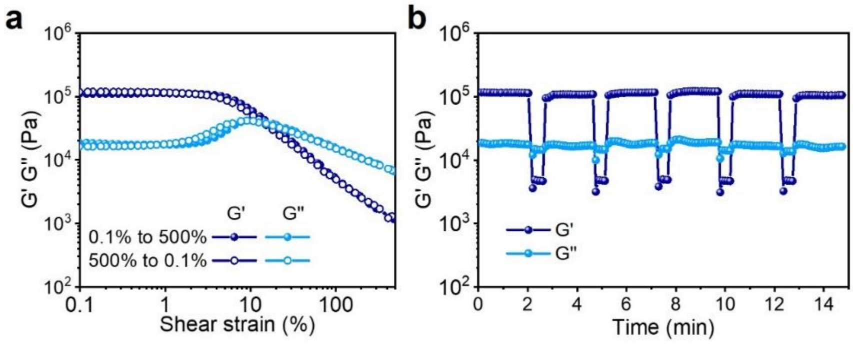

Fig. S3 (**a**) The oscillatory measurements (1 Hz) of the precursor ink (Alg-Mellitic-4), which sweep from 0.1% to 500% and back to 0.1% strain. (**b**) Continuous step-strain oscillatory measurements from 0.1% to 100% and back to 0.1% strain

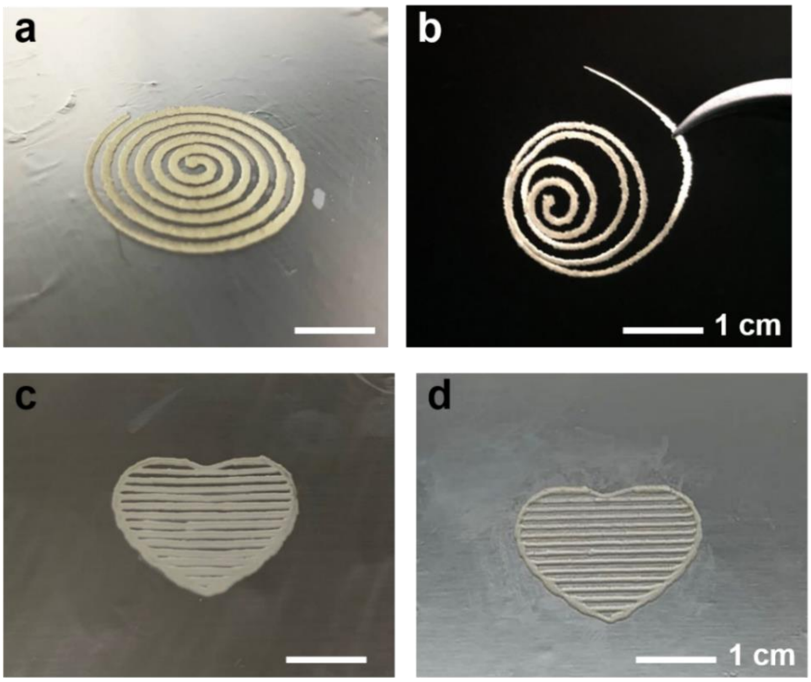

Fig. S4 (**a**) Digital photos of 3D printed spiral coil and (**b**) photo after in situ growth of TbMOFs. (**c**) Digital photo of 3D printed heart and (**d**) photo after in situ growth of EuMOFs

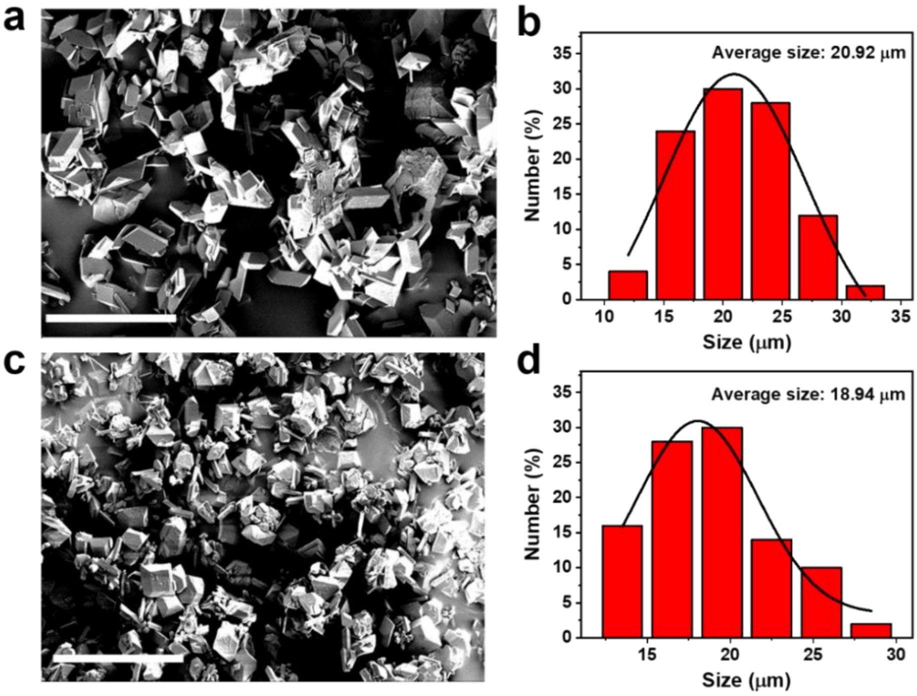

Fig. S5 a, b SEM images and size distribution of EuMOFs with ethanol as precipitant agents (Scale bar = 100 μm). **c, d** SEM images and size distribution of TbMOFs with ethanol as precipitant agents (Scale bar = 100 μm)

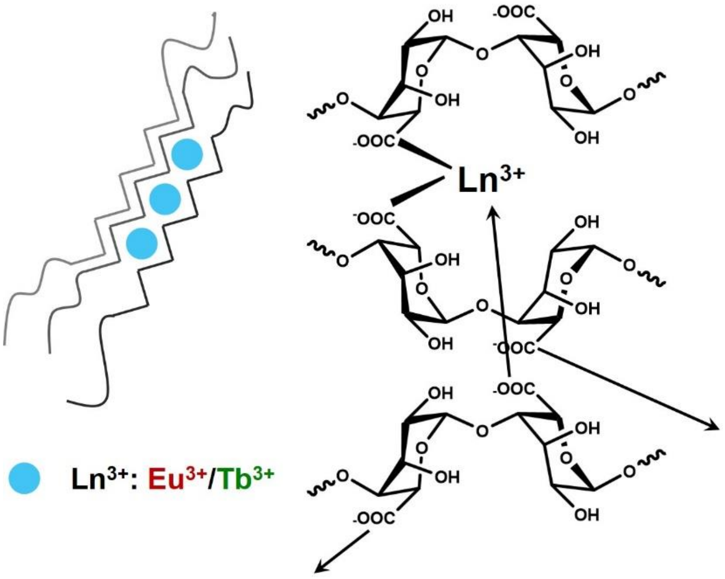

Fig. S6 Schematic diagram of crosslink between trivalent lanthanide ions and sodium alginate

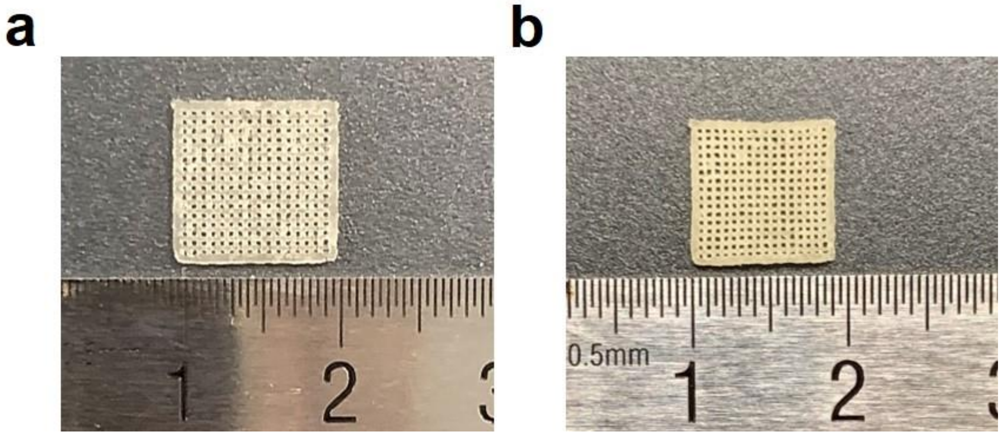

Fig. S7 (**a**) 3D printed scaffold (length: 10.5 mm) with the precursor ink of Alg-Mellitic-4 and (**b**) after immersion in the lanthanide ions solutions (length: 9 mm)

Note: The scaffold was printed with the smallest diameter muzzle (0.26 mm) and the pressure of 0.58 MPa based on the existing 3D printing system. When subsequently subjected the 3D scaffold into the lanthanide ions for the *in-situ* growth of LnMOFs and crosslink with alginate, the volume shrank evenly, further improved resolution (>260 μm). The volume shrinkage of the prints mainly occurs when immersed in metal solutions. We consider that there are two main reasons. One is caused by the crosslinking of lanthanide ions with alginate, and solvent exchange with ethanol causes the other. We estimate the volume shrinkage of the prints by measuring the shrinkage of the side length, the length shrinkage is about 14.3% (from 10.5 mm to 9 mm), accordingly, the volume shrinkage is calculated about 37.0%.

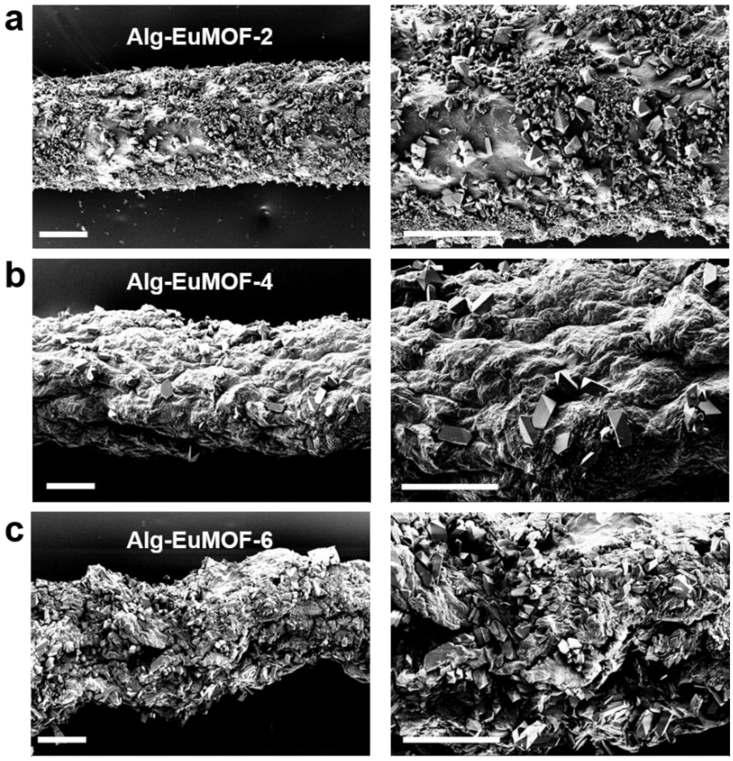

Fig. S8 SEM images of **(a)** Alg-EuMOF-2, **(b)** Alg-EuMOF-4, **(c)** Alg-EuMOF-6 with the post-printing treatment of Eu^{3+}. Scale bars are100 μm

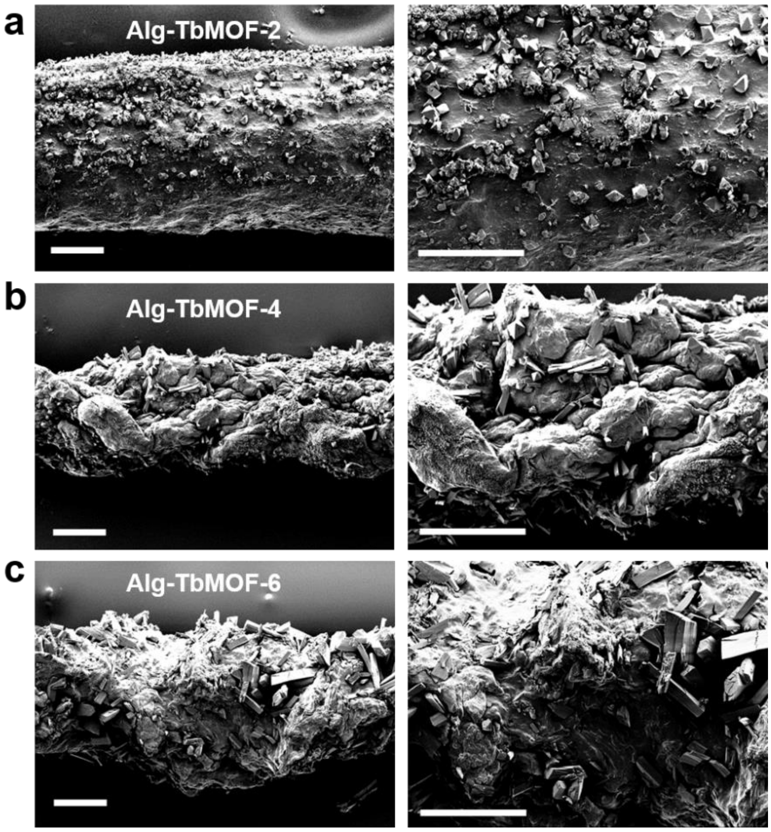

Fig. S9 SEM images of **(a)** Alg-TbMOF-2, **(b)** Alg-TbMOF-4, **(c)** Alg-TbMOF-6 with the post-printing treatment of Tb^{3+}. Scale bars are 100 μm

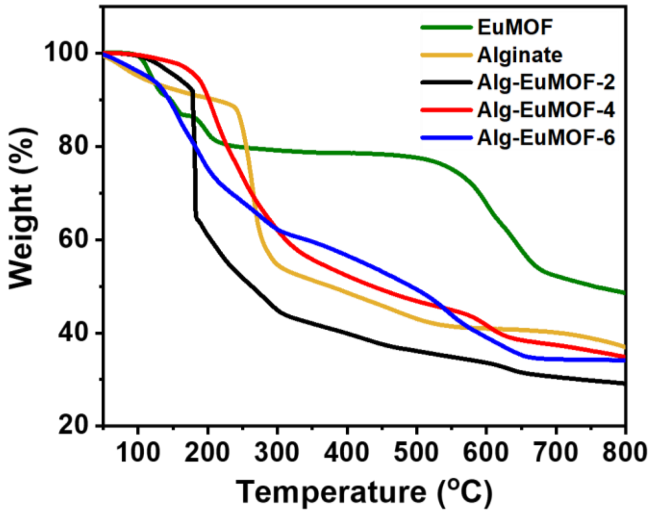

Fig. S10 TGA traces of EuMOF, Alginate, Alg-EuMOF-2, Alg-EuMOF-4, and Alg-EuMOF-6

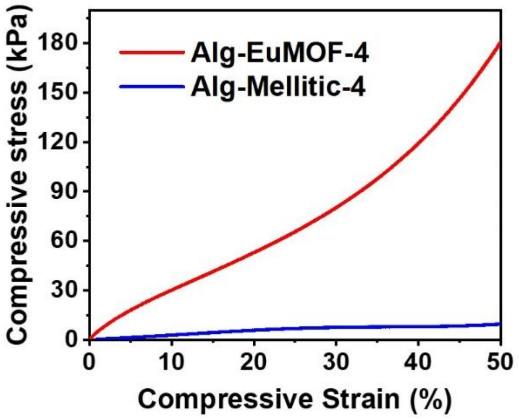

Fig. S11 Mechanical properties of 3D printed scaffold before (Alg-Mellitic-4) and after (Alg-EuMOF-4) immersed in the solution of Eu^{3+} ions

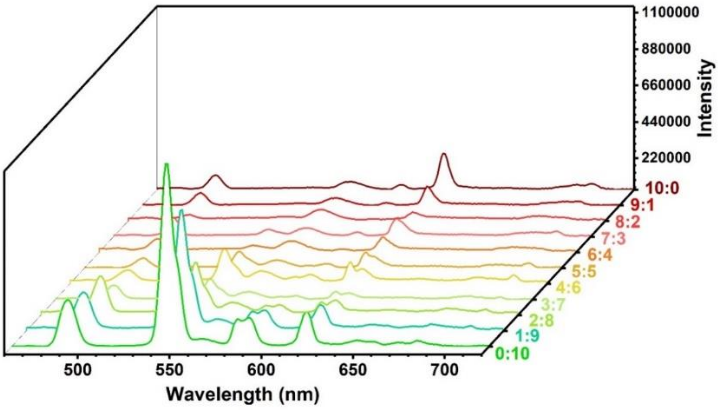

Fig. S12 Luminescent emission spectra of the 3D printed LnMOFs with various molar ratios of Eu^{3+} and Tb^{3+} (λ_{exc} = 254 nm) corresponding to Fig. 4a

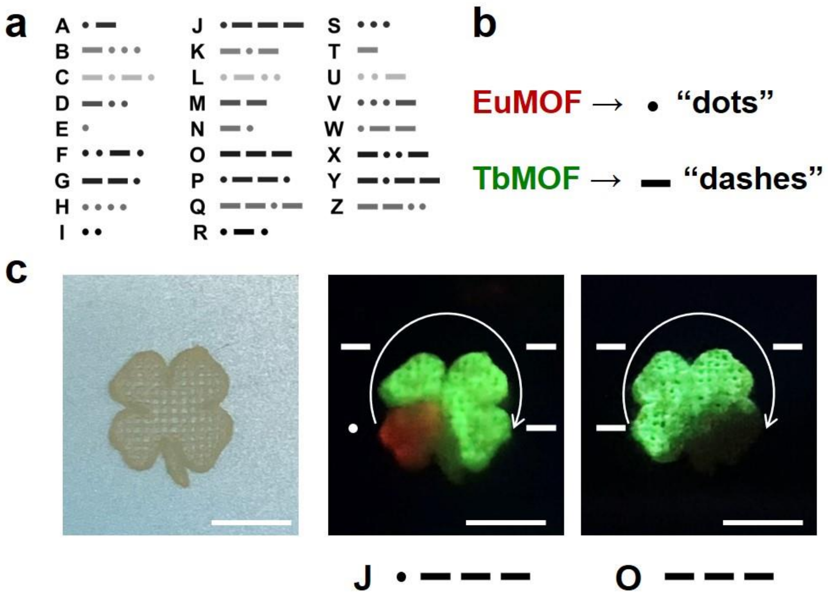

Fig. S13 **(a)** Coding principles of Morse code. (**b**) Illustration of in-situ generated LnMOFs that are used as Morse code encoder. (**c**) Representative encrypted letter examples of "J" and "O"

Note: International Morse code is a character encoding language used in telecommunication that encodes text information as a series of signals called "dots" and "dashes" in various forms such as on-off tones, lights, clicks, or electrical signals (Fig. S13a). In our work, the red and green fluorescent EuMOF and TbMOF can respectively represent "dots" and "dashes" signals, as Fig. S13b shows. Dripping Ln^{3+} and Tb^{3+} ions in a specific sequence can generate specific fluorescent color signals, simple Morse code information such as letters "J" and "O" can be transmitted (Fig. S13c).

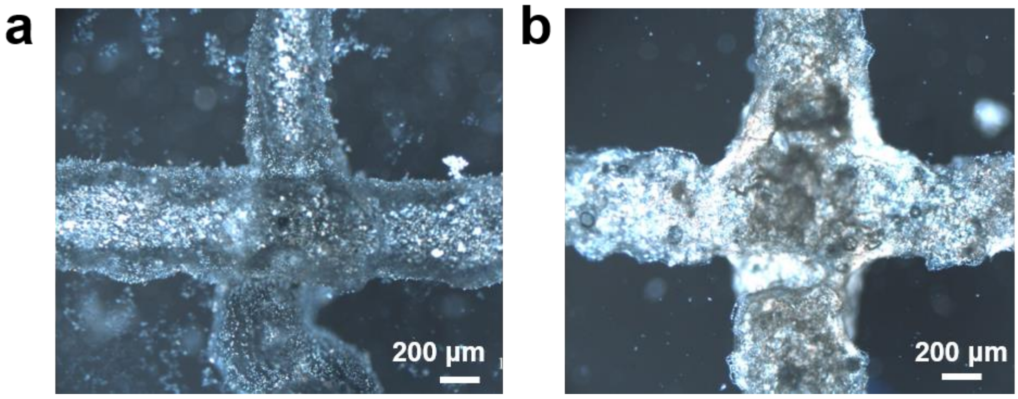

Fig. S14 Photographs of 3D printed structure with the post-printing treatment of mixed metals by polarized optical microscopy (POM)

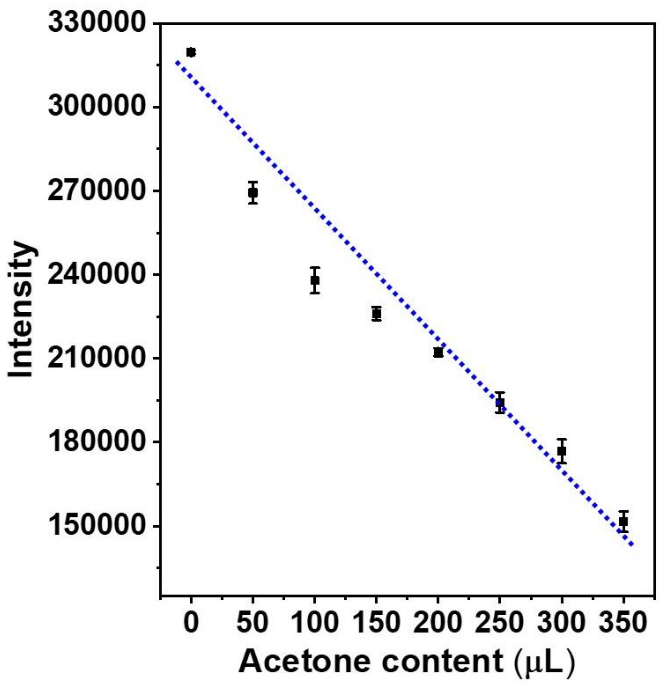

Fig S15 The $^5D_0 \rightarrow {}^7F_2$ transition intensity changes in response to various content acetone among five repetitive experiments (λ_{exc} = 254 nm)

Note: We estimated the limit of detection (LOD) with the linear plot in Fig. S14, as mostly used in sensing evaluation [S1, S2]. Thus, limit of detection = 3σ/slope = (3 x 627) / 490 = 3.83 μL. (σ represents sample standard deviation for the blank without the addition of acetone and is calculated to be 627, sample number = 5).

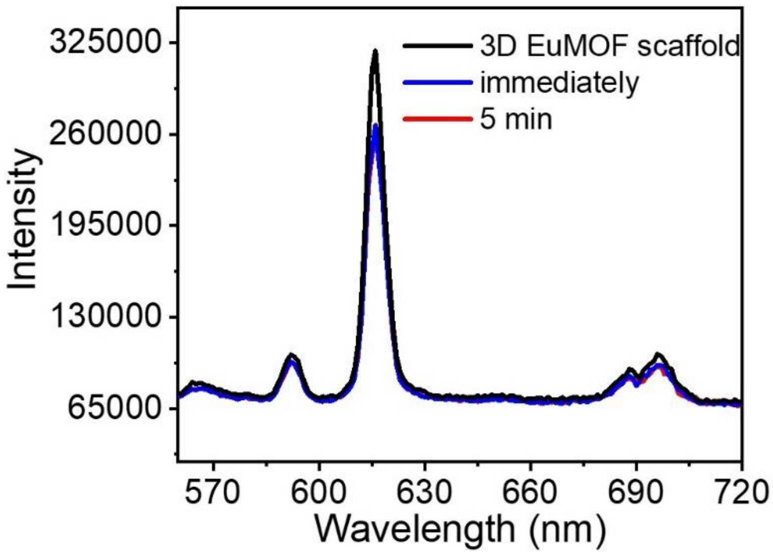

Fig. S16 Time-dependent emission spectra for the 3D EuMOF scaffold in response to acetone (50 μL)

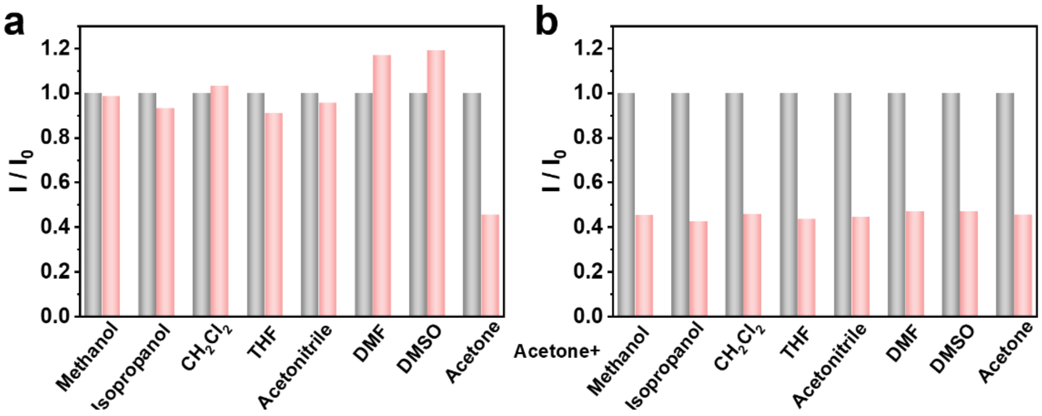

Fig. S17 (**a**) The relative luminescence intensities of the $^5D_0 \rightarrow {}^7F_2$ transition of the 3D scaffold before (grey) and in response to different solvents (red). (**b**) The relative luminescence intensities of the $^5D_0 \rightarrow {}^7F_2$ transition of the 3D scaffold before (grey) and in response to a mixture of 5 vol% other solvent with 95 vol% acetone (red). I and I_0 represent instant emission intensity and original emission intensity, respectively.

Note: When the same content of different solvents was added (including methanol, isopropanol, CH_2Cl_2, THF, acetonitrile, DMF, DMSO and acetone), the intensity of the main transition $^5D_0 \rightarrow {}^7F_2$ is more or less affected by solvent. Particularly, the 3D scaffold exhibited the strongest quenching effect in response to acetone, indicating the selectivity to acetone. We subsequently studied a possible interference of various similar molecules on selective quenching of the luminescence of the sensor caused by acetone. It was found that there is no significant impact of other small amounts of similar molecules (e.g., 5 vol%) on the quenching of the fluorescence caused by acetone.

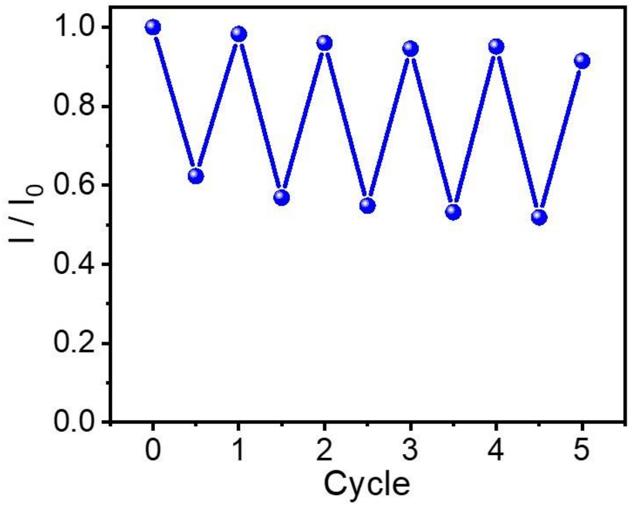

Fig. S18 Relative $^5D_0 \rightarrow {}^7F_2$ transition intensity changes (λ_{ex} = 254 nm) in five quenching-recovery cycles. I and I_0 represent instant emission intensity and original emission intensity, respectively

Table S1 Summary of resolution with works related to 3D printed MOFs

**Ink composition**	**3D printing technology**	**Resolution (μm)**	**Refs.**
Sodium alginate/gelatin/CuBTC	Direct Ink Writing (DIW)	210	[S3]
TOCNF/sodium alginate/ZIF-8	Direct Ink Writing (DIW)	410	[S4]
Silica/PVA/MOF (Ni-BT and ZIF-7)	Direct Ink Writing (DIW)	850	[S5]
PLA/TPU/ZIF-8	Fused Deposition Modeling (FDM)	400	[S6]
Co-kaolin	Direct Ink Writing (DIW)	>700	[S7]
Co-MOF-F127	Direct Ink Writing (DIW)	200	[S8]
Acrylates/UIO-66	Direct Ink Writing (DIW)	>1 mm	[S9]
PVA/Clay/MOF (MOF-74 and UTSA-16)	Direct Ink Writing (DIW)	850	[S10]
Aromatic monomer/crosslinker/ Cu-BTC	Digital Light Processing (DLP)	100	[S11]
Fumed silica/MOF (MIL-100 and HKUST-1)	Direct Ink Writing (DIW)	450	[S12]
ABS/MOF (HKUST-1 and ZIF-8)	Fused Deposition Modeling (FDM)	>700	[S13]
Methylcellulose/ bentonite/ZIF-8	Direct Ink Writing (DIW)	250	[S14, S15]
Nylon-12/HKUST-1	Selective Laser Sintering (SLS)	Not given	[S16]
Torlon/MOF (MOF-74 and HKUST-1)	Direct Ink Writing (DIW)	>500	[S17]
Sodium alginate/mellitic acid (ligand of LnMOFs)	Direct Ink Writing (DIW)	260	This work

Table S2 Comparison of sensing properties for acetone using different sensors related to MOFs

MOFs	Selectivity	Sensitivity	Reversibility	Response rate	Refs.
$[Tb_4(\mu_6\text{-}L)_2(\mu\text{-}HCOO)(\mu_3OH)_3(\mu_3\text{-}O)(DMF)_2(H_2O)_4]_n$	Yes	Not given	Not given	Not given	[S18]
$[Eu_5(DBA)_3]_n$	Yes	Detection limit: 1.24 µM (3mg)	Not given	Not given	[S2]
$[Me_2NH_2]_2[(Eu)_2(ofdp)_2(DMF)(H_2O)]·7H_2O·DMF$	Yes	Detection limit: 7.4 vol%	Not given	Fast	[S19]
MOF-808-Tb	Yes	High (fluorescence intensity reduced to almost 50% with a concentration of below 500 ppm)	Yes	Rapid	[S20]
$[Tb(FDA)_{1.5}(DMF)]DMF_n$	Yes	Fluorescent intensity almost disappeared at an acetone content of 14 vol%	Not given	Yes	[S21]
[Cd(dcba)(DMA)]·DMA	Yes	K_{sv}:8.4 × 10^4 M^{-1}	Rapid	Yes	[S22]
DhaTab-COF-EuIL	Yes	Detection limit: 1.0 vol%	Not given	Not given	[S23]
Eu/Tb@Bi-MOF	Yes	Not given	Not given	Not given	[S24]
Eu–BPDA	Yes	Luminescence intensities decreased to 50% (EC_{50}) at an acetone content of 0.028 vol%	Yes	Fast	[S25]
$[Tb(L)_2·2H_2O]·Cl·4H_2O$ (L = 4-carboxy-1-(4-carboxybenzyl)pyridinium)	Yes	K_{sv}:2.0263 × 10^4 M^{-1}	Yes	Not given	[S26]
3D printed EuMOF scaffold	Yes	Limit of detection: 3.83 µL	Yes	Rapid	This work

Movie S1 3D printing process for 2D patterns and 3D scaffolds

Supplementary References

[S1] B. Hu, P. Wu, Facile synthesis of large-area ultrathin two-dimensional supramolecular nanosheets in water. Nano Res. **13**(3), 868-874 (2020). https://doi.org/10.1007/s12274-020-2709-9

[S2] L. Gao, C. Jiao, H. Chai, Y. Ren, G. Zhang et al., A highly sensitive multifunctional eu-mof sensor with pentacarboxylate for fluorescence detecting acetone, Cu^{2+} and $Cr_2O_7^{2-}$, and electrochemical detection of TNP. J. Solid State Chem. **284**, 121199 (2020). https://doi.org/https://doi.org/10.1016/j.jssc.2020.121199

[S3] R. Pei, L. Fan, F. Zhao, J. Xiao, Y. Yang et al., 3d-printed metal-organic frameworks within biocompatible polymers as excellent adsorbents for organic dyes removal. J. Hazard Mater. **384,** 121418 (2019). https://doi.org/https://doi.org/10.1016/j.jhazmat.2019.121418

[S4] S. Sultan, H.N. Abdelhamid, X. Zou, A.P. Mathew, Cellomof: Nanocellulose enabled 3d printing of metal–organic frameworks. Adv. Funct. Mater. **29**(2), 1805372 (2019). https://doi.org/10.1002/adfm.201805372

[S5] H. Thakkar, Q. Al-Naddaf, N. Legion, M. Hovis, A. Krishnamurthy et al., Adsorption of ethane and ethylene over 3d-printed ethane-selective monoliths. ACS Sustainable Chem. Eng. **6**(11), 15228-15237 (2018). https://doi.org/10.1021/acssuschemeng.8b03685

[S6] K.A. Evans, Z.C. Kennedy, B.W. Arey, J.F. Christ, H.T. Schaef et al., Chemically active, porous 3d-printed thermoplastic composites. ACS Appl. Mater. Interfaces **10**(17), 15112-15121 (2018). https://doi.org/10.1021/acsami.7b17565

[S7] S. Lawson, Q. Al-Naddaf, A. Krishnamurthy, M.S. Amour, C. Griffin et al., Utsa-16 growth within 3d-printed co-kaolin monoliths with high selectivity for CO_2/CH_4, CO_2/N_2, and CO_2/H_2 separation. ACS Appl. Mater. Interfaces **10**(22), 19076-19086 (2018). https://doi.org/10.1021/acsami.8b05192

[S8] Z. Lyu, G.J.H. Lim, R. Guo, Z. Kou, T. Wang et al., 3d-printed MOF-derived hierarchically porous frameworks for practical high-energy density Li–O_2 batteries. Adv. Funct. Mater. **29**(1), 1806658 (2019). https://doi.org/10.1002/adfm.201806658

[S9] A.J. Young, R. Guillet-Nicolas, E.S. Marshall, F. Kleitz, A.J. Goodhand et al., Direct ink writing of catalytically active uio-66 polymer composites. Chem. Commun. **55**(15), 2190-2193 (2019). https://doi.org/10.1039/C8CC10018G

[S10] H. Thakkar, S. Eastman, Q. Al-Naddaf, A. A. Rownaghi, F. Rezaei, 3d-printed metal–organic framework monoliths for gas adsorption processes. ACS Appl.

Mater. Interfaces **9**(41), 35908-35916 (2017). https://doi.org/10.1021/acsami.7b11626

[S11] O. Halevi, J.M.R. Tan, P.S. Lee, S. Magdassi, Hydrolytically stable MOF in 3d-printed structures. Adv. Sustainable Syst. **2**(2), 1700150 (2018). https://doi.org/10.1002/adsu.201700150

[S12] D. Liu, P. Jiang, X. Li, J. Liu, L. Zhou et al., 3d printing of metal-organic frameworks decorated hierarchical porous ceramics for high-efficiency catalytic degradation. Chem. Eng. J. **397**, 125392 (2020). https://doi.org/https://doi.org/10.1016/j.cej.2020.125392

[S13] M. Bible, M. Sefa, J.A. Fedchak, J. Scherschligt, B. Natarajan et al., 3d-printed acrylonitrile butadiene styrene-metal organic framework composite materials and their gas storage properties. 3D Print Addit. Manuf. **5**(1), 63-72 (2018). https://doi.org/10.1089/3dp.2017.0067

[S14] J. Lefevere, B. Claessens, S. Mullens, G. Baron, J. Cousin-Saint-Remi, J.F.M. Denayer, 3d-printed zeolitic imidazolate framework structures for adsorptive separations. ACS Appl. Nano Mater. **2**(8), 4991-4999 (2019). https://doi.org/10.1021/acsanm.9b00934

[S15] B. Claessens, N. Dubois, J. Lefevere, S. Mullens, J. Cousin-Saint-Remi, J.F.M. Denayer, 3d-printed ZIF-8 monoliths for biobutanol recovery. Ind. Eng. Chem. Res. **59**(18), 8813-8824 (2020). https://doi.org/10.1021/acs.iecr.0c00453

[S16] E. Lahtinen, R.L.M. Precker, M. Lahtinen, E. Hey-Hawkins, M. Haukka, Selective laser sintering of metal-organic frameworks: Production of highly porous filters by 3d printing onto a polymeric matrix. ChemPlusChem **84**(2), 222-225 (2019). https://doi.org/10.1002/cplu.201900081

[S17] S. Lawson, M. Snarzyk, D. Hanify, A.A. Rownaghi, F. Rezaei, Development of 3d-printed polymer-MOF monoliths for CO_2 adsorption. Ind. Eng. Chem. Res. **59**(15), 7151-7160 (2020). https://doi.org/10.1021/acs.iecr.9b05445

[S18] Q. Zhang, J. Wang, A.M. Kirillov, W. Dou, C. Xu et al., Multifunctional ln–mof luminescent probe for efficient sensing of Fe^{3+}, Ce^{3+}, and acetone. ACS Appl. Mater. Interfaces **10**(28), 23976-23986 (2018). https://doi.org/10.1021/acsami.8b06103

[S19] Y. Yang, L. Chen, F. Jiang, M. Wu, J. Pang et al., A water-stable 3d Eu-MOF based on a metallacyclodimeric secondary building unit for sensitive fluorescent detection of acetone molecules. CrystEngComm **21**(2), 321-328 (2019). https://doi.org/10.1039/C8CE01875H

[S20] J. Zhang, S.B. Peh, J. Wang, Y. Du, S. Xi et al., Hybrid MOF-808-Tb nanospheres for highly sensitive and selective detection of acetone vapor and Fe^{3+} in aqueous solution. Chem. Commun. **55**(32), 4727-4730 (2019). https://doi.org/10.1039/C9CC00178F

[S21] H. Li, W. Shi, K. Zhao, Z. Niu, H. Li, P. Cheng, Highly selective sorption and luminescent sensing of small molecules demonstrated in a multifunctional lanthanide microporous metal–organic framework containing 1d honeycomb-type channels. Chem. Eur. J. **19**(10), 3358-3365 (2013). https://doi.org/10.1002/chem.201203487

[S22] J. Wang, J. Wang, J. Wu, Y. Li, L. Zheng, Y.-Y. Lin, A luminescent Cd(ii)-MOF based on flexible biimidazolyl-benzenecarboxylate ligand for selectively sensing of acetone. Inorg. Chem. Commun. 108167 (2020). https://doi.org/https://doi.org/10.1016/j.inoche.2020.108167

[S23] H. Zuo, Y. Li, Y. Liao. Europium ionic liquid grafted covalent organic framework with dual luminescence emissions as sensitive and selective acetone sensor. ACS Appl. Mater. Interfaces **11**(42), 39201-39208 (2019). https://doi.org/10.1021/acsami.9b14795

[S24] L. Xu, Y. Xu, X. Li, Z. Wang, T. Sun, X. Zhang, Eu^{3+}/Tb^{3+} functionalized bi-based metal–organic frameworks toward tunable white-light emission and fluorescence sensing applications. Dalton Trans. **47**(46), 16696-16703 (2018). https://doi.org/10.1039/C8DT03474E

[S25] J. Wang, J. Wang, Y. Li, M. Jiang, L. Zhang, P. Wu, A europium(iii)-based metal–organic framework as a naked-eye and fast response luminescence sensor for acetone and ferric iron. New J. Chem. **40**(10), 8600-8606 (2016). https://doi.org/10.1039/C6NJ02163H

[S26] K. Wang, Y. Ma, H. Tang, Lanthanide coordination polymers as luminescent sensors for the selective and recyclable detection of acetone. Crystals **7**(7), 199 (2017). https://doi.org/10.3390/cryst7070199
